# Global trends in social-ecological systems and disaster research: Bibliometric insights

**DOI:** 10.4102/jamba.v17i1.1874

**Published:** 2025-09-30

**Authors:** Simon S. Hutagalung, Yulianto Yulianto, Feni Rosalia

**Affiliations:** 1 Department of Development Studies, Faculty of Social and Political Studies, University of Lampung, Bandar, Indonesia; 2 Department of Public Administration, Faculty of Social and Political Studies, University of Lampung, Bandar, Indonesia; 3 Department of Government Studies, Faculty of Social and Political Studies, University of Lampung, Indonesia

**Keywords:** social-ecological systems, disaster, bibliometric analysis, disaster risk reduction, climate adaptation

## Abstract

Understanding the dynamics of social-ecological systems (SES) in the context of disasters is essential to bridge the interactions among humans, the environment and disaster risks. This perspective supports the development of disaster risk reduction (DRR) strategies and the strengthening of community resilience while addressing practical and scientific gaps in the literature. The present study analysed global research trends on SES and disasters, focusing on publication patterns, thematic clusters and the geographical distribution of contributions. It employed a bibliometric analysis of 140 Scopus-indexed papers retrieved with relevant keywords. Network visualisation and data-density techniques were applied to identify research trends, thematic groupings and regional participation. The results highlight emerging themes and recurrent topics within SES and disaster studies, with substantial contributions from the United States, China, Germany and Australia and a steady annual increase in publications. Major thematic clusters included DRR, nature conservation, disaster management and climate adaptation. Areas gaining greater attention were climate adaptation, DRR and ecosystem management, signalling shifting priorities in SES and disaster research. This study underscores adaptation, resilience, governance and policy as central themes and emphasises the importance of interdisciplinary approaches and expanded databases for future investigations.

**Contribution:**

The research provides a comprehensive overview of SES and disaster trends, offering insights to policymakers and practitioners to enhance DRR strategies and global resilience initiatives. It fills a critical gap in understanding the interplay between SES dynamics and disaster management.

## Introduction

Social-ecological systems (SES) in the context of disasters pertain to the intricate and ever-changing interconnections among humans, the natural environment and disasters (Crawford 2020). A social system comprises individuals, communities and institutions that interact and function collectively to form the structure and dynamics of society (Biggs et al. 2021). Social systems play a critical role in shaping how humans interact with the environment and how they respond to, and are affected by, disasters. In contrast, ecological systems encompass all living and non-living components of the environment – including plants, animals, water, soil and air – that sustain life and provide essential resources for human well-being (Hossain et al. 2024). Disasters – commonly defined as serious disruptions to the functioning of a community or society involving widespread human, material, economic or environmental losses and impacts that exceed the affected community’s or society’s ability to cope using its resources – can exert a substantial influence on both social and natural systems (United Nations Office for Disaster Risk Reduction [UNDRR] 2020). Such events often harm human populations, infrastructure and ecosystems (Depietri 2020; Zimmerman, Willig & Hernández-Delgado 2020) significantly.

The interaction between human societies and the natural environment is most evident in activities such as agriculture, fishing and the extraction of natural resources. While these practices are essential for sustaining livelihoods, they also have profound implications for the environment. Rather than directly driving climate change in all cases, these activities often exacerbate its impacts. For example, deforestation for agricultural expansion reduces carbon sequestration capacity, while certain farming practices and fossil fuel-dependent resource extraction contribute to greenhouse gas emissions. As climate change leads to more frequent and intense weather-related events – such as storms, floods and droughts – the vulnerabilities created or intensified by unsustainable human activities can amplify the adverse effects on both ecosystems and human communities. In this way, the consequences of environmental degradation become more severe in the context of climate change, which, in turn, increases the frequency and intensity of hazard events like floods and droughts. Research on SES emphasises the interconnectedness between human and ecological components, acknowledging that while human societies depend on the environment, the reverse is not necessarily true. These studies underscore that disasters can significantly disrupt human systems and ecological processes. Understanding the role of SES – defined as integrated systems in which human and natural components interact dynamically – is essential for developing effective strategies to reduce disaster risks and strengthen long-term resilience (Biggs et al. 2021). By comprehending the interconnectedness between human societies and the natural environment, we can deepen our understanding of the drivers and mechanisms that contribute to disaster events. This insight is essential for developing more effective policies and strategies aimed at reducing disaster risk, strengthening preparedness and facilitating post-disaster recovery and rehabilitation (Castillo et al. 2020). In disaster studies, key concepts include hazards (natural or anthropogenic triggers of disasters), exposure (the presence of people or assets in hazard-prone areas) and vulnerability (susceptibility to harm). These interact to define disaster risk (UNDRR 2023). The SES framework integrates ecological and social system interactions to better understand and address disaster risks, including their underlying drivers and potential impacts.

The SES framework provides an integrated lens to analyse the complex interactions between human societies and ecological processes in disaster-prone contexts. Rather than suggesting that an SES itself is designed or capable of deliberate action, the framework is used to understand how spatial planning, environmental management and social behaviour contribute to disaster risk and resilience (Folke et al. 2010; Ostrom 2009). For instance, locating infrastructure – such as housing, roads and water systems – in areas with lower exposure to hazards like flooding is a common risk reduction strategy. However, such structural interventions must be grounded in a sound understanding of local hydrology and ecological systems. Poorly designed infrastructure, particularly flood control projects that disregard the complexity of watershed dynamics, can paradoxically increase disaster risks (UNDRR 2017). In contrast, non-structural approaches – such as reforestation to prevent erosion and landslides or community education programmes that enhance awareness and preparedness – focus on behaviour change, institutional capacity and ecosystem-based adaptation. According to the UNDRR (2017), structural measures involve physical constructions to reduce hazard exposure, while non-structural measures include policy, knowledge and practices that aim to modify human behaviour and reduce vulnerability. By applying the SES framework, decision-makers can better identify the social, ecological and institutional drivers of disaster risk. This enables more context-sensitive strategies that integrate both structural and non-structural measures, ultimately enhancing adaptive capacity and strengthening community resilience in the face of increasing disaster threats (Zimmerman et al. 2020).

Existing disaster research that adopts an SES approach has primarily focused on natural hazards at the local or community level (Talubo, Morse & Saroj 2022). Examples include community resilience to wildfire (Prior & Eriksen 2013), disaster as an external shock in the context of the Indian Ocean tsunami of 2004 (Renaud et al. 2010) and the impact of climate change on fishing livelihoods (Salgueiro-Otero & Ojea 2020). Most disaster studies focus on area-based case studies because of the context-specific nature of SES (Berkes & Folke 1998; Turner et al. 2003). While these provide in-depth insights, they often lack broader applicability. Broader-scale SES research is needed to address transboundary disasters and identify cross-scalar patterns for adaptive governance (Cumming 2011; Ostrom 2009). Given the increasing frequency and severity of hazard events – driven by climate change and anthropogenic pressures – broader-scale SES research is urgently needed to inform systemic, cross-regional resilience strategies. Understanding the relationships between SES and disasters is crucial for developing resilience and mitigating the risks associated with hazard events and their underlying drivers (Li, Dong & Liu 2020; Saja et al. 2021).

An SES is a dynamic system shaped by interactions between human societies and the natural environment. Understanding these interactions is vital for disaster risk reduction (DRR) and resilience-building. While academic research enhances this understanding, publications alone are insufficient for broader impact. To inform policy and practice, research must also be translated into accessible formats such as policy briefs, toolkits and public engagement efforts, ensuring uptake beyond academic circles (Biggs et al. 2021; Castillo et al. 2020). Effectively managing disaster risk requires a multidisciplinary approach that integrates perspectives from the social sciences, natural sciences and engineering disciplines. Although scientific publications have the potential to facilitate cross-disciplinary communication, in reality, scholars often remain within the boundaries of their own disciplinary literature. As such, genuine interdisciplinary collaboration is more likely to emerge through structured mechanisms such as co-authored research, interdisciplinary research consortia and integrated methodological frameworks that actively promote dialogue and knowledge exchange across disciplinary lines. Publication alone is insufficient to ensure meaningful engagement between disciplines (Rutting et al. 2022).

While academic publications contribute to understanding SES and disasters, broader research outputs – such as community engagement and knowledge co-production – are essential to translate findings into effective policies and resilience strategies beyond academic audiences (Folke et al. 2021). Scientific papers serve as an important medium for disseminating findings from research on SES and disasters to those involved in policymaking and professional practice. Advancing our understanding of SES and disaster dynamics through scholarly publications is essential for informing decision-making processes and promoting evidence-based resilience strategies. The theoretical basis of this study draws on the Sendai Framework for DRR (UNDRR 2015), which emphasises understanding disaster risk, strengthening governance and investing in resilience. The SES framework is rooted in Ostrom’s (2009) work on coupled human–environment systems, emphasising complexity, feedback loops and adaptation capacity (Folke et al. 2021).

The primary aim of this study is to provide a comprehensive overview of the current research landscape on SES in the context of disasters by analysing academic publication data. Through bibliometric analysis, the study seeks to map the intellectual structure of the field, identify key themes and highlight knowledge gaps to help prioritise future research efforts. Such an approach supports more informed and strategic directions in DRR (Rana 2020). Bibliometric methods offer valuable insights by revealing dominant topics, emerging trends and underrepresented regions in SES and disaster scholarship. Understanding these patterns is increasingly vital as the frequency and intensity of extreme weather events and other hazards continue to rise. To guide the analysis, this study addresses the following three research questions: (1) What are the global research trends in SES and disaster studies? (2) What topics and themes are most frequently investigated in the literature? and (3) How have research patterns in this field evolved over time and across different regions? By answering these questions, the study aims to identify gaps and trends in the existing literature on SES in disaster contexts, thereby providing a foundation for future academic inquiry. While not directly informing policy or practice, the findings can help shape future research agendas that may, in turn, support evidence-based approaches to disaster resilience.

## Research methods and design

This study employed a bibliometric analysis to investigate research trends in the field of SES and disasters. Bibliometric analysis is a quantitative method used to evaluate scientific literature by analysing bibliographic data such as article titles, authors, journals, publication dates and citation counts (Agarwal et al. 2016). This method measures research productivity, scientific impact and trends in a research field using bibliographic data, such as the article title, author, journal, date and citations (Alsharif, Salleh & Baharun 2020). This method facilitates the identification of dominant research themes, emerging trends and existing gaps in the literature, thereby providing valuable insights for scholars, librarians and policymakers. The research process began with the formulation of precise and well-defined research questions aimed at uncovering the intellectual structure and publication patterns of SES and disaster research. To answer these questions, a comprehensive literature search was conducted across multiple sources, including major academic databases such as Scopus and Dimension. This multi-source approach was intended to ensure broad coverage of relevant scholarly work.

Given the technical requirements of bibliometric tools, the analysis was intentionally limited to peer-reviewed journal articles indexed in Scopus and Dimension, because they offer structured metadata suitable for this type of analysis. While this methodological decision enhances the consistency and reliability of the data, it is acknowledged that important insights on DRR are also found in grey literature – such as reports from the UNDRR, the International Federation of Red Cross and Red Crescent Societies (IFRC) and the Global Network of Civil Society Organisations for Disaster Reduction (GNDR). Future studies should consider incorporating these non-academic sources to achieve a more comprehensive understanding of the field.

To ensure relevance and data quality, specific keywords and search filters were applied. Keywords were selected through an iterative process that combined prior literature review with expert domain knowledge. The primary search terms included ‘social-ecological systems’, ‘disaster’, ‘resilience’ and ‘climate adaptation’. The search was further refined using Scopus filters: document type was limited to journal articles (LIMIT-TO (DOCTYPE, ‘ar’)), source type to journals (LIMIT-TO (SRCTYPE, ‘j’)) and language to English (LIMIT-TO (LANGUAGE, ‘English’)). These parameters ensured a consistent dataset comprising peer-reviewed articles that explicitly addressed SES dynamics within disaster contexts.

**FIGURE 1:**
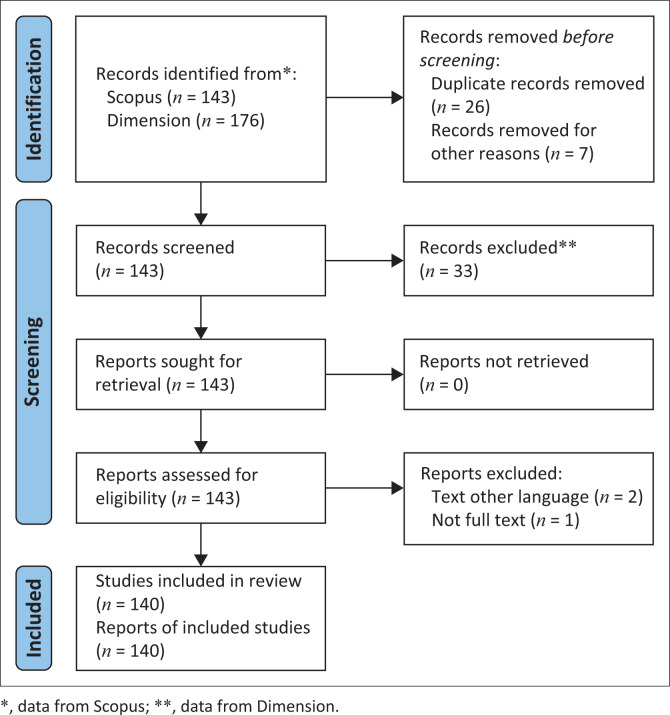
Preferred Reporting Items for Systematic Reviews and Meta-Analyses diagram for socio-ecological system and disaster topics.

Following the initial retrieval of records, a systematic screening process was conducted using the PRISMA (Preferred Reporting Items for Systematic Reviews and Meta-Analyses) framework. A total of 143 articles were retrieved from Scopus and 176 from Dimension.. After removing 26 duplicate records and excluding seven articles because of irrelevance or incomplete metadata, 286 records were screened. Following this, 33 articles were excluded based on the inclusion criteria – mainly because of language constraints (2 articles) and lack of full-text access (1 article). In the end, 140 articles met the eligibility criteria and were included in the final analysis. The PRISMA flow diagram for this bibliometric study is presented in Figure 1.

Subsequently, a data cleaning process was undertaken. This involved standardising the formatting of bibliographic entries (e.g. author names, publication year, journal title), correcting typographical errors and ensuring consistency across the dataset. Key terms were extracted from article titles, abstracts and keyword fields to facilitate thematic analysis. The cleaned dataset was then analysed using both descriptive and network-based techniques. Descriptive analyses examined temporal trends based on publication years, the frequency of keyword occurrences, the number of publications per journal and country-level contributions. Journals were considered ‘leading’ based on the number of articles published on the topic. In addition, network analyses were conducted to visualise institutional collaborations and keyword co-occurrence patterns, helping to identify key thematic clusters and areas of active research.

### Ethical considerations

This article followed all ethical standards for research without direct contact with human or animal subjects.

## Results and discussion

This section presents a temporal analysis of articles related to SES and disasters, structured around the year of publication to explore patterns in research activity over time. The analysis covers 19 years from 2006 to 2024. This time frame was selected because 2006 marks the earliest publication on this topic indexed in the Scopus database, while 2024 represents the most recent year available at the time of data collection. The year of publication was used as the basis for this analysis, as it provides a standardised metric across all articles. However, it is acknowledged that publication dates do not necessarily reflect the timing of the research itself. Because of peer review and editorial processes, there is often a significant lag between the completion of a study, its submission and eventual publication (Bornmann & Daniel 2008). Therefore, the trends identified in this section reflect the dissemination of research findings rather than the precise timing of the original investigations. By analysing the number of publications per year, the study identifies temporal trends in scholarly interest in SES and disaster-related topics, including years with the highest and lowest output. This approach also identifies emerging research areas or subthemes, providing insights into how the focus of SES and disaster research has evolved.

Comparing research activity by country or region helps identify global patterns in SES and disaster research output, particularly the institutions and countries where studies are conducted or published. However, many studies conducted in lower-income regions are led by scholars from developed countries, which may skew perceptions of local research capacity (Gaillard 2019). This analysis focuses only on English-language publications indexed in Scopus, thereby excluding non-English and locally published research, especially from regions like South America and Asia. These limitations should be considered when interpreting the geographic distribution of publications. The publication trends presented in Table 1 offer further insight into the evolution and direction of research on SES and disasters.

**TABLE 1:** Distribution of publications by year.

No.	Year	Quantity	%
1	2024	12	8.57
2	2023	17	12.14
3	2022	13	9.29
4	2021	17	12.14
5	2020	4	2.86
6	2019	7	5.00
7	2018	12	8.57
8	2017	11	7.86
9	2016	7	5.00
10	2015	10	7.14
11	2014	10	7.14
12	2013	6	4.29
13	2012	4	2.86
14	2011	6	4.29
15	2010	3	2.14
16	2006	1	0.71

**Total**	**-**	**140**	**-**

Overall, the data demonstrate a growing academic interest in the topic, with a notable increase in publications after 2020, peaking in 2021 and 2023, each contributing 12.14% of total publications, while earlier years, such as 2006, show minimal activity, suggesting a relatively recent surge in scholarly engagement. The temporal distribution of publications shows a general upward trend in scholarly interest in the intersection of SES and disasters, particularly in the post-2020 period. While annual fluctuations are visible, a notable rise is observed in 2023, suggesting renewed academic focus on this topic following the COVID-19 pandemic. Such variations may reflect broader dynamics within the research ecosystem. For example, global disruptions like pandemics can momentarily suppress academic productivity because of shifting institutional priorities and limited field access (Else 2020). Conversely, increased attention to socio-ecological resilience in the aftermath of major disasters or policy shifts may stimulate publication surges (Birkmann 2006; Folke 2016). Internal institutional factors – such as the availability of research funding, interdisciplinary collaboration and the presence of dedicated research – also shape the pace and output of scientific publications. Thus, understanding these publication trends provides insight into the evolving priorities and capacities of the global research community addressing SES and disaster risk.

Analysing authorship patterns in SES and disaster research provides insight into the structure and diversity of scholarly contributions. Based on the 140 documents identified in this bibliometric analysis, approximately 20.2% were authored by researchers who contributed more than one publication within this specific dataset on SES and disasters. This means that some authors appeared multiple times in the corpus, indicating sustained research interest in the topic. The remaining 79.8% of the publications were single contributions by authors who appeared only once in the dataset. This observation was derived from an analysis of author names across the 140 selected documents, not from a full review of all papers published by each author since 2006. This distribution reflects the diversity and openness of the SES and disaster research landscape, with a majority of contributors engaging with the topic through individual publications, while a smaller group of researchers shows sustained scholarly engagement. This suggests a highly diverse and decentralised research field with many contributors entering the discourse, often from different disciplinary backgrounds. Although no author dominates the field, Renaud and Tidball, who come from related disciplines within the SES field, stand out with four publications each. Renaud focuses on environmental risk and resilience, while Tidball is rooted in environmental psychology and community-based resource management. While this number is modest in absolute terms, their repeated engagement may indicate a sustained research interest and potential influence in shaping the direction of SES-disaster scholarship. Institutional factors may also play a role – authors affiliated with well-funded research focused on resilience or environmental governance may have more opportunities to publish. However, this analysis did not assess co-authorship networks or institutional affiliations in detail. Existing literature on bibliometric methodologies emphasises the need for caution in interpreting authorship frequency as a proxy for scholarly impact, especially without citation analysis or contextual factors (Aria & Cuccurullo 2017; Donthu et al. 2021).

Analysing the distribution of publications by country in the context of SES and disasters offers insights into global research capacity, collaboration patterns and geographic disparities in knowledge production. This information can be useful for academic institutions, research funders and international organisations – such as UNDRR or UNEP – that design global frameworks for DRR and sustainability. For example, identifying leading countries or emerging contributors may help shape future research partnerships and capacity-building efforts. The data show that the United States (US) leads in publication output, followed by China, Germany, Australia and the United Kingdom (UK). While the US remains dominant throughout the period covered in the analysis, its share has slightly declined during this time, whereas China has shown a marked rise, becoming the second-most productive country in terms of publications on SES in the context of disasters by 2023. This trend reflects broader structural inequalities in academic publishing, where countries with more resources are better positioned to publish in high-impact international journals. Publication fees, language barriers and limited research infrastructure often hinder participation from scholars in lower-income countries, despite their direct exposure to disaster impacts.

The distribution of publications is influenced by factors such as the maturity of academic disciplines, national research priorities and exposure to disasters. Countries such as the US, the UK and Germany lead in publication output, largely because of their strong research infrastructures and sustained investment in the environmental and social sciences (Aria & Cuccurullo 2017). Nations with high exposure to natural hazards – such as Japan and the US – also demonstrate significant research productivity in disaster-related fields. This observation aligns with global disaster risk assessments provided by the UNDRR (2022). In addition, government-funded research initiatives, such as the European Union’s Horizon 2020 programme, illustrate how policy frameworks and strategic funding priorities can drive scholarly output by incentivising interdisciplinary, cross-national collaboration in fields like disaster resilience and SES. However, this analysis is limited to English-language publications in Scopus and Dimension. Research from disaster-prone developing countries, such as Indonesia or the Philippines, may be underrepresented because of publication in local or non-indexed journals and possibly in other languages (Gaillard 2010).

Analysing the distribution of publications by institutional affiliation in the context of SES and disasters provides valuable insights into the global research landscape, key contributing institutions and emerging collaboration networks. This type of bibliometric analysis can support a range of stakeholders – including academic researchers, university administrators, funding agencies and policymakers – by helping them identify leading institutions, monitor research output and allocate resources strategically. For example, funding bodies can use such analyses to target institutions or regions with lower research output for capacity-building initiatives. Policymakers may also refer to bibliometric data to understand which institutions are producing influential knowledge in areas such as disaster resilience or climate adaptation. Previous studies (e.g. Aria & Cuccurullo 2017; Van Eck & Waltman 2014) have shown how bibliometric mapping has informed national research strategies, identified innovation hubs and guided science policy decisions, particularly in fields related to sustainability and DRR.

The publication data reveal active contributions from a range of universities and research centres, with Stockholm University (eight publications) and the Stockholm Resilience Centre (seven publications) leading the list, both institutions known for their focus on resilience and SES. Other key institutions include Cornell University, the Chinese Academy of Sciences and Beijing Normal University. These organisations host specialised departments or centres that support research in SES and DRR. A notable portion of publications also comes from 33 institutions categorised as ‘Other’, each contributing three papers. This reflects broad institutional participation and, in some cases, international collaboration. For example, Biggs et al. (2012) and Berkes and Ross (2013) feature co-authors from multiple institutions across countries, indicating cross-institutional efforts. The higher output from institutions in countries like Sweden, the US and China can be attributed to stronger research infrastructure, funding and access to relevant data.

Analysing publication sources in SES and disaster research helps reveal where key knowledge is produced and how it circulates. This information benefits researchers (in choosing journals), editors (in assessing field positioning) and policymakers or funders (in identifying research gaps and priority areas). Such bibliometric insights support informed decisions in research strategy and policy (Aria & Cuccurullo 2017; Van Raan 2005). Based on the data, the distribution of publications shows that research on SES and disasters is published in various sources. The source with the most publications is *Ecology and Society* (15 publications), followed by *Sustainability Switzerland* (8 publications) and the *International Journal of Disaster Risk Reduction* (5 publications). Ecology and Society appears to concentrate on the intersection of ecology and social systems. It often publishes interdisciplinary research that examines the complex relationships between humans and the natural environment. The journal *Sustainability Switzerland* focuses on a wide range of sustainability-related issues and frequently publishes research with a regional emphasis on Switzerland and the broader European context, as evidenced by the geographical distribution of contributing authors and case studies featured in its publications. The journal *Sustainability* publishes research on a broad spectrum of topics related to environmental and social sustainability, including renewable energy, climate change mitigation and sustainable development. In contrast, the *International Journal of Disaster Risk Reduction* is specifically dedicated to advancing knowledge in DRR, encompassing areas such as disaster preparedness, emergency response, post-disaster recovery and risk prevention. Other sources identified in this bibliometric dataset contribute fewer publications on the topic and exhibit a more limited thematic focus. The concentration of SES and disaster-related research in certain journals reveals where scholarly discourse is most active. Identifying these core publication venues helps researchers and practitioners target relevant outlets and understand the disciplinary orientations that shape the field. Although this analysis reflects only a portion of the broader literature, it provides valuable insight into how knowledge is organised and disseminated across disciplines, supporting more strategic engagement in this interdisciplinary domain.

To gain a more comprehensive understanding of the intersection between SES and disasters – as well as to map the broader research landscape – it is useful to examine the 10 most highly cited articles in this domain. The insights derived from this analysis can serve as a valuable reference for researchers, practitioners and policymakers aiming to enhance knowledge development and disaster preparedness within SES frameworks. Table 2 presents a summary of the top 10 most cited publications addressing the linkages between SES and disasters:

**TABLE 2:** Top 10 publications on socio-ecological and disaster topics.

No	Authors	Title	Year	Source title	Cited by
1	Adger W.N.	Vulnerability	2006	Global Environmental Change	4110
2	Zhou H., Wang J., Wan J., Jia H.	Resilience to natural hazards: A geographic perspective	2010	Natural Hazards	346
3	Adger W.N., Brown K., Nelson D.R., Berkes F., Eakin H., Folke C., et al.	Resilience implications of policy responses to climate change	2011	Wiley Interdisciplinary Reviews: Climate Change	273
4	Carpenter S.R., Arrow K.J., Barrett S., Biggs R., Brock W.A., Crépin A.-S., Engström G., Folke C., et al.	General resilience to cope with extreme events	2012	Sustainability	270
5	Ungar M.	Systemic resilience: principles and processes for a science of change in contexts of adversity	2018	Ecology and Society	207
6	Kotzee I., Reyers B.	Piloting a social-ecological index for measuring flood resilience: A composite index approach	2016	Ecological Indicators	195
7	Carey M., Huggel C., Bury J., Portocarrero C., Haeberli W.	An integrated socio-environmental framework for glacier hazard management and climate change adaptation: Lessons from Lake 513, Cordillera Blanca, Peru	2012	Climatic Change	181
8	Matin N., Forrester J., Ensor J.	What is equitable resilience?	2018	World Development	161
9	Lorenz D.F.	The diversity of resilience: Contributions from a social science perspective	2013	Natural Hazards	159
10	Ferraro P.J., Sanchirico J.N., Smith M.D.	Causal inference in coupled human and natural systems	2019	Proceedings of the National Academy of Sciences of the United States of America	146

Note: Please see the full reference list of this article, Hutagalung, S.S., Yulianto, Y. & Rosalia, F., 2025, ‘Global trends in social-ecological systems and disaster research: Bibliometric insights’, *Jàmbá: Journal of Disaster Risk Studies* 17(1), a1874. https://doi.org/10.4102/jamba.v17i1.1874, for more information.

The data reflect an expanding body of research on SES and disasters, with several notable trends. Firstly, there is an increasing focus on resilience, particularly in understanding how SES can adapt to and recover from disruptions such as climate change and disasters (Adger 2006; Adger et al. 2011; Carpenter et al. 2012; Ungar 2018; Zhou et al. 2010). Secondly, a growing number of studies emphasise the integration of social and ecological dimensions, marking a shift towards more holistic approaches in disaster research (Carey et al. 2012; Kotzee & Reyers 2016). Thirdly, the concept of equitable resilience has emerged, highlighting the need to address social justice and inequality in resilience-building efforts (Matin, Forrester & Ensor 2018). Lastly, advances in methodology, including the use of causal inference in studying coupled human–natural systems, signal a move towards more rigorous and nuanced SES analysis (Ferraro, Sanchirico & Smith 2019). Causal inference in coupled human and natural systems demonstrates progress in developing robust methods for studying SES dynamics.

### Keyword cluster analysis in the topic of socio-ecological systems and disasters

Keyword cluster analysis is a method commonly used in bibliometric analysis to identify main themes in publication data sets (Wang, Zhao & Wang 2018). Research trends and patterns can be better understood, as well as the structure and content of data sets, with the use of this method. Table 3 presents the results of cluster and keyword analysis performed on the subject of SES and disasters.

**TABLE 3:** Identified clusters and keywords.

No	Cluster	Keywords
1	Cluster 1	Adaptive management, climate adaptation, coastal zone, coastal zone management, decision-making, disaster management, disaster risk reduction, extreme event, governance, governance approach, nature-society relations, resilience, risk assessment, stakeholder, sustainability, vulnerability
2	Cluster 2	Conservation of natural disasters, ecosystem resilience, environmental protection, flooding, humans, seashore, simulation
3	Cluster 3	Disaster prevention, drought, ecology, flood, hazard assessment, interdisciplinary approach, natural hazards, social-ecological system
4	Cluster 4	Adaptation, climate change, ecosystem service, ecosystem, environmental change, fisheries, natural disasters, resource management, socioeconomic, sustainable development

Based on the bibliometric keyword clustering, four major thematic areas emerge, reflecting the dominant research directions in the field of SES and disaster studies. The first cluster focuses on DRR, particularly in coastal zones. Keywords such as *disaster risk reduction, climate adaptation, coastal zone management* and *resilience* suggest a strong emphasis on proactive strategies to reduce vulnerability and enhance sustainability in the face of climate-related hazards. The second cluster centres on nature conservation, highlighting themes such as *biodiversity, ecosystem services* and *environmental protection*. This indicates a growing concern with preserving ecological integrity and the critical role of ecosystems in mitigating disaster risks. The third cluster relates to disaster management, with frequent terms including *flood, risk assessment, GIS* and *extreme events*. This cluster reflects practical and technological approaches to managing and understanding disaster impacts, particularly hydrometeorological hazards. The fourth cluster addresses climate adaptation, with key terms such as *climate resilience, resource management* and *adaptation policy*. This theme captures the increasing focus on long-term strategies to enhance adaptive capacity across ecological and social systems. Together, these clusters offer a comprehensive overview of current research priorities, supporting a more integrated understanding of disaster risk, environmental sustainability and adaptive governance.

The bibliometric keyword clustering reveals strong thematic interconnections between DRR, nature conservation, disaster management and climate adaptation. This convergence underscores the need for integrated, interdisciplinary approaches to address the multifaceted challenges posed by climate change and disaster risk. Key research themes emerging from the analysis include climate adaptation, resilience-building, sustainability and policy development – each critical for enhancing the capacity of communities and ecosystems to withstand and recover from disruptions. The findings highlight the importance of cross-sectoral collaboration among disciplines such as ecology, sociology, economics and public policy. A holistic research agenda grounded in these intersections can inform more effective and lasting solutions. Moreover, the identification of these themes provides strategic direction for future inquiry, supporting efforts to foster resilience and sustainability in the face of growing socio-environmental risks.

### Network visualisation analysis, overlay and density

**FIGURE 2:**
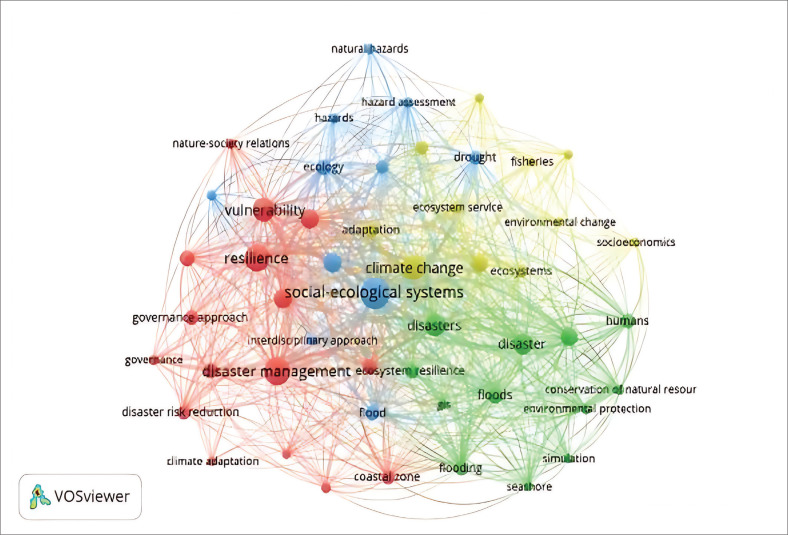
Network visualisation on socio-ecological and disaster topics.

Network visualisation, conducted in this study using VOSviewer, maps co-occurrence patterns among keywords to reveal the structural relationships within SES and disaster research. This method identifies central themes – such as resilience, governance and climate change – and highlights underexplored areas through weakly connected or peripheral nodes (Parlina, Ramli & Murfi 2020). By visualising these connections, the analysis helps uncover knowledge gaps, suggests directions for future research and reveals potential avenues for interdisciplinary collaboration (Mazandarani & Royo-Vela 2022). While primarily used for academic analysis, network visualisation can also inform policy and communication by clarifying research priorities. To improve interpretability, future studies should present higher-resolution figures with detailed legends. Figure 2 depicts the visualisation of the publication network on the topic of SES and disasters.

Figure 2 illustrates the thematic structure of SES and disaster research through network visualisation. Key trends include an increasing focus on society–environment interactions, with terms such as *vulnerability, biodiversity* and *environmental change* closely linked, reflecting recognition that both ecological processes and human actions shape disasters. Clusters around *adaptation, resilience* and *climate change* highlight the shift towards proactive, systems-based approaches. Governance and policy also emerge as central themes, underscoring their role in shaping disaster outcomes. Lastly, terms related to specific hazards – such as floods and droughts – are often embedded within broader socio-environmental contexts, reinforcing the widely accepted view that disasters are not purely natural events but are socially constructed and shaped by human vulnerabilities and decisions (Cutter 2006; Kelman 2020; Wisner et al. 2004). This perspective emphasises the role of anthropogenic factors – such as land use, governance and inequality – in influencing disaster impacts.

The network visualisation analysis highlights several core research areas within the field of SES and disaster studies. Firstly, there is a strong emphasis on the interaction between society and the environment, reflecting scholarly interest in how socio-environmental systems respond to and influence disaster risk. Secondly, themes of adaptability and resilience are prominent, particularly regarding how communities and ecosystems adjust to the impacts of climate change and compounding hazards. Thirdly, governance and policy emerge as key nodes in the network, indicating growing attention to institutional frameworks and their role in reducing vulnerability and enhancing resilience. Lastly, hazard-specific studies – such as those addressing floods, droughts and coastal risks – are situated within broader socio-ecological contexts, recognising that such events are not purely natural but are shaped by human actions and systemic conditions. Overall, the network visualisation provides a comprehensive overview of the evolving research landscape, underscoring the interdisciplinary and integrative nature of contemporary disaster scholarship.

**FIGURE 3:**
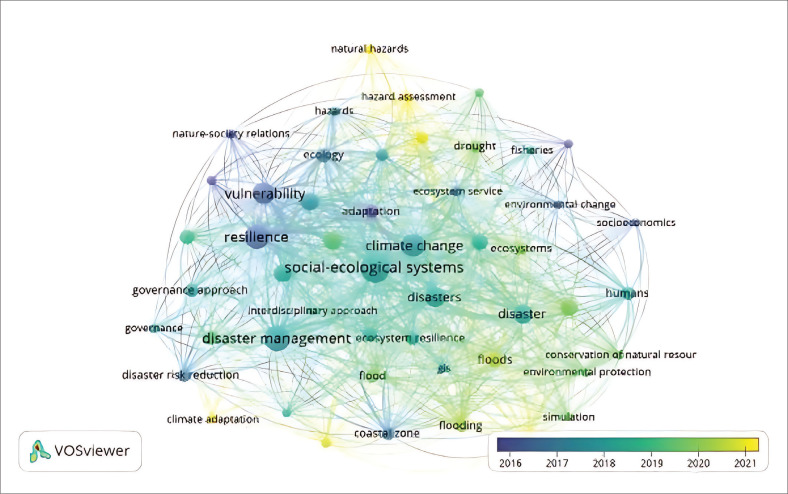
Overlay visualisation publications on socio-ecological and disaster topics.

Overlay visualisation in bibliometric analysis is a method that maps research themes or keywords while illustrating their temporal progression, often using colour gradients to indicate average publication years. This allows researchers to identify emerging trends, shifts in focus and underexplored areas within a field (Raza, Govindaluri & Bhutta 2022). In the context of SES and disaster studies, overlay visualisation highlights how concepts like resilience and governance have gained prominence over time while revealing less developed areas that may warrant further investigation. Such insights can inform future research priorities and support evidence-based decision-making in policy and practice (Chien et al. 2019). Overlay visualisation of bibliometric data can help facilitate collaboration between researchers working in the same or related fields. By visualising relationships between researchers, an overlay visualisation of bibliometric data can help identify opportunities for collaboration and joint research. Figure 3 presents an overlay visualisation of the topic of SES and disasters.

The overlay visualisation highlights the temporal evolution of research topics in the field of SES and disasters through a colour-coded mapping. In Figure 3, dark blue indicates early research concentrations on natural disasters, including earthquakes, floods and droughts. Light blue represents studies focused on climate change adaptation, such as disaster-resilient infrastructure and early warning systems. Green denotes themes related to SES, including sustainable resource management and community resilience, while yellow reflects research on emergency preparedness and disaster management.

The visualisation in Figure 3 suggests that between 2006 and 2013, natural disasters dominated scholarly attention. From 2014 to 2021, there has been a notable shift towards climate change adaptation, accompanied by a growing emphasis on the resilience of SES and risk management. These trends indicate an expanding research agenda that moves beyond reactive disaster response to encompass more integrated and anticipatory approaches. Overall, the overlay visualisation demonstrates how the field has evolved in both focus and complexity, reflecting a broader interdisciplinary engagement with sustainability, governance and adaptive capacity.

**FIGURE 4:**
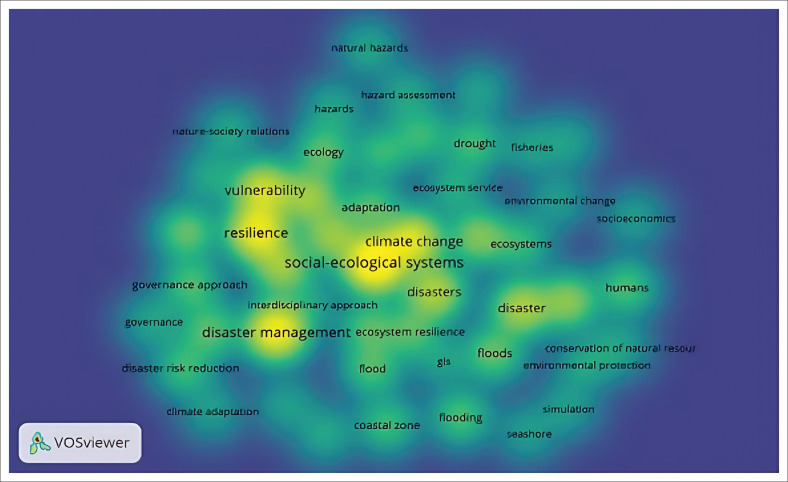
Density visualisation on socio-ecological systems and disaster topics.

Scopus bibliometric data density visualisation helps analyse research trends and landscapes, detect knowledge gaps and track research progress (Ezugwu et al. 2021). Analysis helps academics, politicians and the public make better judgements in many domains. Figure 4 shows the density visualisation analysis results.

The bibliometric density visualisation illustrates the distribution and intensity of research focus across the SES and disaster literature. As expected, the term ‘social-ecological systems’ features prominently in the analysis, reflecting the initial keyword selection criteria. While this prominence affirms the relevance of SES as a conceptual lens in disaster-related research, it also highlights a limitation of bibliometric methods: findings are necessarily shaped by the initial search parameters. Consequently, themes not explicitly captured by the selected keywords – such as biodiversity, indigenous knowledge systems, ecosystem services, or cultural heritage – may be underrepresented in the results despite their substantive relevance to SES thinking. Nevertheless, the density visualisation reveals several broad thematic areas that warrant further reflection. Firstly, there is a discernible concentration of studies engaging with the sustainability and management of coupled human–environment systems, underscoring the growing interest in integrated approaches to socio-environmental governance. Secondly, governance and policy frameworks emerge as focal points, aligning with the global policy landscape shaped by the Sustainable Development Goals (SDGs) and international climate agreements, such as the Paris Agreement. These frameworks increasingly emphasise the need for systemic, cross-sectoral interventions to build resilience and reduce vulnerability (UN 2015; UNFCCC 2016). This reflects a growing recognition in the literature of governance as a central mechanism in addressing complex socio-ecological challenges (Jordan et al. 2010; Lebel et al. 2006). Thirdly, science and technology – such as modelling and decision-support tools – frequently appear in the literature, suggesting a shift towards data-informed SES analysis. However, this may reflect the search focus, which excluded terms related to traditional or indigenous knowledge, potentially underrepresenting non-technological approaches. While the current visualisation reflects the dominant topics within the search-defined dataset, it also implicitly indicates the need to expand future bibliometric analyses by incorporating a broader range of terms. This approach would enable the identification of additional facets of SES research, such as cultural traditions, indigenous knowledge systems and localised ecological understandings – components that are often integral to resilience yet tend to be underrepresented in conventional scholarly literature.

### Summary of the key findings

A network analysis of publications on SES and disasters reveals several interesting trends. Research has become more focused on the interactions between nature and society, as well as the importance of adaptation and resilience in the face of disasters. The role of governance and policy in reducing disaster risk is also increasingly recognised. In addition, research covers a variety of disasters, indicating the need to understand a wide range of threats. Overlay analysis shows a shift in research focus over time, from natural disasters to climate change adaptation, SES resilience and risk management. Meanwhile, density analysis highlights the importance of SES and the role of science and technology in their management. These findings have significant implications for research, policy and disaster management practices, emphasising the importance of an interdisciplinary approach, collaboration and the use of data visualisation.

A network analysis of publications – utilising network visualisation, overlay mapping and density analysis – has yielded valuable insights into the evolving structure and thematic complexity of research on SES and disasters. Network visualisation elucidates the interconnections among key concepts such as natural hazards, adaptation, resilience and governance. Overlay analysis illustrates the temporal shifts in research focus, while density analysis highlights the concentration of scholarly attention on particular themes and domains. Collectively, these analytical approaches demonstrate that SES-disaster research has become increasingly interdisciplinary, integrating perspectives from environmental science, policy studies and social systems analysis. For researchers, such findings help to identify underexplored areas, inform future research directions and facilitate collaboration across disciplines. For disaster management practitioners, the identified thematic clusters may serve as a basis for developing more integrated and context-sensitive strategies. While the findings of this analysis may also hold relevance for policymakers, it is important to acknowledge the structural barriers to accessing academic knowledge. Most scholarly publications remain behind paywalls, limiting their accessibility to individuals not affiliated with academic institutions. As a result, the potential impact of academic research on policy development depends significantly on the extent to which findings are synthesised and disseminated through accessible channels, such as open-access publications, policy briefs, or practitioner-oriented reports. Bridging the gap between research and policy, therefore, necessitates targeted knowledge translation efforts to ensure that academic insights reach and inform the policy community effectively.

The observed trends and thematic landscapes identified in this study are not only relevant for advancing academic understanding but also hold potential implications for policy and practice within the field of DRR. By systematically mapping underexplored research areas and emerging themes, the findings offer a foundation for identifying directions in which further scholarly inquiry and practical innovation may be warranted. Rather than presenting prescriptive recommendations, this study highlights analytical patterns within the literature that may support the development of more context-aware policies and programmes aimed at mitigating disaster risks and enhancing community resilience. In this way, the study contributes to the broader discourse on the role of scientific knowledge in informing sustainable and adaptive responses to complex socio-environmental challenges. This study is limited by its focus on peer-reviewed, English-language journal articles indexed in Scopus. As a result, contributions from non-English-speaking scholars or grey literature may be underrepresented. Future studies should incorporate multilingual databases and reports from global institutions to enrich the findings.

## Conclusion

Research on SES and disasters has shown a significant upward trend in recent years, reflecting growing awareness of the intricate linkages between human societies and natural systems in disaster contexts. This study has provided a systematic overview of the field by mapping the thematic landscape and publication patterns through bibliometric analysis. The results reveal a shift from reactive approaches focused on disaster response towards more integrated strategies that emphasise prevention, mitigation, adaptation and resilience. Core themes emerging from the analysis include climate adaptation, DRR, governance and nature conservation. These themes point to an increasing convergence of disciplines and signal a broader understanding of the social-ecological dimensions of disaster risk. Research output remains concentrated in a few high-income countries, indicating the need for more inclusive and geographically diverse contributions. An important finding of this study is the growing emphasis on governance and institutional mechanisms within SES-disaster research. This trend reflects the increasing integration of SES thinking into national DRR frameworks and policy agendas. By identifying dominant themes and emerging gaps, this study offers insights for policymakers, particularly in highlighting underexplored areas of risk, shifting resilience narratives and evolving institutional priorities. The prominence of ‘governance’ and ‘climate adaptation’ in recent research clusters demonstrates this transition and the need for more systemic approaches to disaster planning and response.

Looking ahead, future bibliometric research could benefit from incorporating broader search terms to capture well-established but underrepresented areas – such as regional case studies, adaptive decision-making tools, policy coherence and the roles of social behaviour, education and community participation in disaster contexts. While these themes are already well documented in the wider literature, they were not fully reflected in this analysis because of the limitations of the initial keyword selection. Ultimately, this study contributes to a clearer understanding of how SES research can inform evidence-based strategies for disaster management. It reinforces the value of interdisciplinary approaches and long-term planning in building more resilient and adaptive societies. By mapping dominant themes and recent trends, this study offers insights into how SES research intersects with evolving DRR priorities. For instance, the prominence of terms like ‘governance’ and ‘climate adaptation’ in recent clusters suggests a growing alignment between SES thinking and national policy discourse. However, given that the keyword search did not include terms related to traditional knowledge or alternative framings, the analysis may not fully capture the diversity of perspectives in the broader literature.
